# Inhibition of the Inflammasome Activity of NLRP3 Attenuates HDM-Induced Allergic Asthma

**DOI:** 10.3389/fimmu.2021.718779

**Published:** 2021-08-03

**Authors:** Ming Ma, Guoyang Li, Minghui Qi, Wei Jiang, Rongbin Zhou

**Affiliations:** ^1^Hefei National Laboratory for Physical Sciences at Microscale, The Chinese Academy of Sciences Key Laboratory of Innate Immunity and Chronic Disease, School of Basic Medical Sciences, Division of Life Sciences and Medicine, University of Science and Technology of China, Hefei, China; ^2^Chinese Academy of Sciences Centre for Excellence in Cell and Molecular Biology, University of Science and Technology of China, Hefei, China

**Keywords:** asthma, inflammasome, HDM, alveolar macrophage, RRx-001, NLRP3

## Abstract

Inhaled allergens promote inflammatory response, tissue damage, and airway hyperresponsiveness in the lungs, leading to allergic asthma. NLRP3, as an immune sensor of infections and cellular stress, is associated with the development and exacerbation of asthma. However, the mechanism by which NLRP3 affects asthma requires further investigation. Here, we showed that inhaled house dust mite (HDM) promotes NLRP3 inflammasome activation in the lungs and specifically induces the maturation of caspase-1 and IL-1β in alveolar macrophages (AMs). Using *Nlrp3*-mutant mice, we found that NLRP3 promotes the inflammatory response and pathogenesis in HDM-induced allergic asthma in an inflammasome-dependent manner. Treatment with RRx-001, an NLRP3 inhibitor, significantly reduced inflammatory cell infiltration and mucus secretion in the airway. Our results showed that NLRP3 in myeloid cells promoted the development and progression of allergic asthma in an inflammasome-dependent manner. Small molecules targeting the NLRP3 inflammasome may provide new treatment options for this disease.

## Introduction

The airway can come into contact with pathogens, infectious agents, and danger signals present in the environment (1, 2). The type II immune response induced by inhaled allergens, infectious agents or air pollution leads to IL-5, IL-4 and IL-13 secretion in the airway, which can lead to eosinophil accumulation, mast cell activation and airway hyperreactivity in allergic asthma (3–5). It has been demonstrated that the recognition of various stimuli and the associated inflammatory responses are important in the development and exacerbation of asthma (6, 7). Corticosteroids, β2 agonists and leukotriene receptor antagonists are commonly used in the treatment of asthma (8). Side effects, including respiratory tract infections and decreased bone density, often occur with the use of inhaled corticosteroids, which are primary medications in all steps of asthma therapy (9, 10). Thus, it is important to find new targets in asthma and to provide new treatments with fewer side effects.

By forming the inflammasome complex, NLRP3 functions as a cellular sensor that is involved in many diseases, such as type 2 diabetes, Alzheimer’s disease, inflammatory bowel disease and some infectious diseases (11, 12). The NLRP3 inflammasome can be activated by infections and danger signals released by tissue damage, leading to cell pyroptosis and IL-1β secretion (13). Several inhibitors of the NLRP3 inflammasome have been reported to prevent tissue destruction in NLRP3-related diseases in animal models (14); thus, NLRP3 is a potential therapeutic target for these diseases (15). NLRP3 also promotes the inflammatory response in allergic asthma (16–19). However, the mechanism by which NLRP3 affects allergic asthma remains unclear (20).

Variants in the *Nlrp3* gene increase the risk of asthma in patients (21). The expression of NLRP3 and caspase-1 in BAL fluids from asthmatic patients is higher than that in BAL fluids from healthy controls (22, 23). The expression of NLRP3 and IL-18 is also increased in the airway epithelium of asthmatic patients compared with healthy controls (24). In an OVA-induced asthma model, allergen inhalation induced ROS production, NLRP3 inflammasome components expression and inflammasome assembly in mouse bronchial epithelial cells (22, 25–27). Another allergen, HDM, has been reported to promote IL-1β secretion in macrophages isolated from asthmatic lungs (28). In addition, it has been demonstrated that NLRP3 in T_H_2 cells promotes the type II immune response in asthma (29). Thus, the cell source of NLRP3 that affects the progression of asthma needs to be investigated.

Although NLRP3 has been identified to promote airway inflammation in allergic asthma, whether the inflammasome activity of NLRP3 alters the progression of asthma is unclear. In the sputum of asthmatic patients, increased IL-1β levels suggest inflammasome activation in the airway (30). In *Nlrp3^-/-^*, *Il-1b^-/-^*, *Asc^-/-^* and *Caspase1^-/-^* mice, the inhaled OVA-induced inflammatory response is reduced, indicating that activation of the NLRP3 inflammasome promotes the development of allergic asthma (17, 18). Inhibition of the NLRP3 inflammasome by neutralizing anti-IL-1β antibody or MCC950 treatment suppresses inflammation and goblet hyperplasia in the airway (22, 25, 31). However, some results suggest that allergen challenge fails to increase the expression of IL-1β and caspase-1 in the airway (25). NLRP3 can act as a transcriptional regulator to induce the differentiation of T_H_2 cells to promote asthma symptoms in an inflammasome-independent manner (29). NLRP3 has been reported to regulate M2 macrophage polarization through interaction with IRF4 and the upregulation of IL-4 in asthma (32). Therefore, the role of the NLRP3 inflammasome in the regulation of asthma also remains unknown.

In this study, we found that HDM challenge induces NLRP3 inflammasome activation in alveolar macrophages. NLRP3 promoted airway inflammation and pathogenesis in an inflammasome-dependent manner in HDM-induced asthma. The covalent NLRP3 inhibitor RRx-001, a well-tolerated agent without clinically significant toxic effects currently in phase III clinical trials, had a significant therapeutic effect on mice challenged with HDMs. Thus, we determined that the NLRP3 inflammasome promoted the inflammatory response in asthma and could be a target for asthma therapy.

## Materials And Methods

### Mice

C57BL/6J mice were obtained from the Laboratory Animal Center of University of Science and Technology of China. *Nlrp3^-/-^* mice and NLRP3^Y30E^ mutant (*Nlrp3^Y30E/Y30E^*) mice were described previously (33, 34). All animals were SPF, housed under a 12 light/12 dark cycle at 20-22°C, with water and food accessible at all times. All of the mouse experiments were approved by the Animal Care Committee of University of Science and Technology of China.

### Reagents

HDM was from Greer Laboratories. Anti-mouse IL-1β (p17) (AF-401-NA) was from R&D. Anti-mouse caspase-1 (p20) (AG-20B-0042) and anti-NLRP3 (AG-20B-0014) antibodies were from Adipogen. Anti-ASC (67824) antibody was from Cell Signaling Technology. Anti-β-actin (66009-1-Ig) was from Proteintech.

Anti-mouse antibodies used for flow cytometry were: CD3-FITC (BD, 553062, 145-2C11), CD19-FITC (eBioscience, 11-0193-82, 1D3), Ly-6G-PE (Biolegend, 127608, 1A8), CD45-PE (eBioscience, 12-0451-81, 30-F11), CD11c-PerCP-Cy5.5 (Biolegend, 117328, N418), CD11b-PerCP-Cy5.5 (BD, 550993, M1/70), CD11b-PE-Cy7 (eBioscience, 25-0112-82, M1/70), CD3e-PE-Cy7 (BD, 552774, 145-2C11), CD19-PE-Cy7 (Biolegend, 115520, 6D5), SiglecF-Alexa Fluor 647 (BD, 562680, E50-2440), MHCII-APC-eFluor 780 (eBioscience, 47-5321-82, M5/114.15.2), Ly-6G-BV421 (Biolegend, 127628, 1A8), CD45-BV510 (Biolegend, 103137, 30-F11), CD11c-BV510 (Biolegend, 117338, N418). Ultrapure LPS was obtained from Invitrogen. Nigericin was obtained from Sigma-Aldrich. RRx-001 (S8405) was from Selleck.

### Mouse Asthma Model

Wild type, *Nlrp3^-/-^*, *Nlrp3^Y30E/Y30E^* mice were anesthetized by intraperitoneal injection of pentobarbital sodium and instilled i.n. with 10μg HDM in 40μl saline on day 0. For days 7-11, mice were challenged daily with 10μg HDM in 40μl saline. Mice were euthanized for analysis on day 14. Mice in the control group were instilled with 40μL saline on day 0 and days 7-11.

### ELISA

Supernatants from BALF, serum, tissue lysates and cell culture were assayed for mouse IL-1β, IL-5, IL-13 or IL-6 (R&D Systems) according to the manufacturer’s instructions. Supernatants from BALF and cell culture were assayed for mouse IL-4 and IFN-γ (Novus) according to the manufacturer’s instructions. Mice serum was assayed for total IgE (BD) according to the manufacturer’s instructions. For HDM-specific IgE measurement, 5μg HDM was coated for each well overnight at 4°C. Then the plate was washed and conjugated with mice serum for 2h at room temperature. The remaining steps were the same as the manufacturer’s instructions of mouse IgE ELISA kit (BD).

### Quantitative PCR With Reverse Transcription

Total RNA of mice lung tissue was isolated with TRIzol (Takara). RNA was applied to generate cDNA using the RevertAid H Minus First Strand cDNA Synthesis Kit (ThermoFisher) according to the manufacturer’s instructions. Quantitative PCR was performed using the Universal SYBR Green Fast qPCR Mix (Abclonal) in a LightCycler 96 Real-Time PCR System (Roche). *Gapdh* was used as a reference gene. The sequences of primers were as follows:

mouse Gapdh forward, GGTGAAGGTCGGTGTGAACG;
mouse Gapdh reverse, CTCGCTCCTGGAAGATGGTG;
mouse Nlrp3 forward, CGAAGCAATGCCCTTGGAGA;
mouse Nlrp3 reverse, GGTGAGGCTGCAGTTGTCTA;
mouse Aim2 forward, ACGTTGTTAAGAGAGCCAGGG;
mouse Aim2 reverse, TTGTCTCCTTCCTCGCACTT;
mouse Nlrc4 forward, CTGTGTGAGCAGTGACGGAT;
mouse Nlrc4 reverse, GCCCGTGAGCTTTACCTCTT;
mouse Mefv forward, GCTTGTGAAGGAAGGTCACAG;
mouse Mefv reverse, AGCATATGGAGCATCTGCCG;
mouse Nlrp1b forward, CAAGCCAGAGGGACACTTGA;
mouse Nlrp1b reverse, ATGGGTACCTCTGTTTGGGC;
mouse IL1b forward, TGCCACCTTTTGACAGTGATG;
mouse IL1b reverse, AAGGTCCACGGGAAAGACAC.

### Analysis of BALF

Mice were euthanized after HDM or saline treatment. Cut the skin on the neck and chest carefully with surgical scissors, cut open the chest cavity to connect to the atmosphere. Expose the trachea, cut a small opening in the trachea and insert the gavage needle to inject 1mL PBS containing 5mM EDTA into the lungs. After 1min filling, the BALF was absorbed by an 1mL syringe. Then repeat the perfusion and aspiration 3 times without waiting for filling. The supernatants of the first BALF were used to measure cytokines. All BALF cells were collected for FACS analyses.

### Stimulation of MLN Cells

After mice were euthanized, the MLN was isolated and ground into single cells suspensions. For one sample, 1x10^6^ MLN cells were cultured at 37°C under 5% CO2 in RPMI 1640 medium supplemented with 10% FBS, 1mM sodium pyruvate, 2mM L-glutamine and 50μg/mL HDM for 5 days. The supernatants were collected after 5-day-culture to detect the cytokines released by MLN cells.

### Flow Cytometry and Cell Sorting

After the 14-d HDM treatment, the mice were euthanized and the lung tissue was removed after perfusion with PBS, cut into small pieces using scissors and digested in 400U/mL collagenase I (Sigma-Aldrich) for 1h in a 37°C shaking incubator (200r.p.m.). The digestion was terminated by diluting with PBS. After lysing red blood cells, the suspension was filtered using a cell strainer (70μM), and then stained with fluorochrome-conjugated antibodies for 30 min. Washed antibody-marked cells were analyzed by BD Verse cytometers or sorted by a FACSAria flow cytometer (BD). BALF cells isolation was described before. After the 14-d HDM or saline treatment, the mice were euthanized. The BALF cells were isolated, stained with fluorochrome-conjugated antibodies and analyzed by BD Verse cytometers. For the caspase-1 activity assay, the cells were stained with FAM-FLICA fluorescent probe (Immunochemistry Technologies) for 40min in a 37°C incubator. And then the cells were washed, stained with antibodies and analyzed by flow cytometry. A description of the gating strategy is provided in **Supplementary Figure 1**.

### Histological Analysis

Lungs were excised and fixed in 4% formalin. Then the fixed lungs were paraffin-embedded. They were sectioned and stained with H&E or PAS. The photos were taken by TissueFAXS PLUS (TissueGnostic Gmbh).

### Cell Culture

BMDMs were derived from C57BL6 mice and cultured in 37°C in DMEM containing 10% FBS, 1mM sodium pyruvate, 2mM L-glutamine in the presence of 50ng/mL recombinant M-CSF (R&D Systems). AMs were derived from C57BL6 mice BALF and cultured in a complete medium.

### Statistical Analyses

All values were expressed as mean ± SEM. Unpaired t-test (GraphPad Prism) was used in all experiments. When *P*-value < 0.05, the data were considered significant.

## Results

### Activation of the NLRP3 Inflammasome in HDM-Induced Allergic Asthma

**Figure 1:**
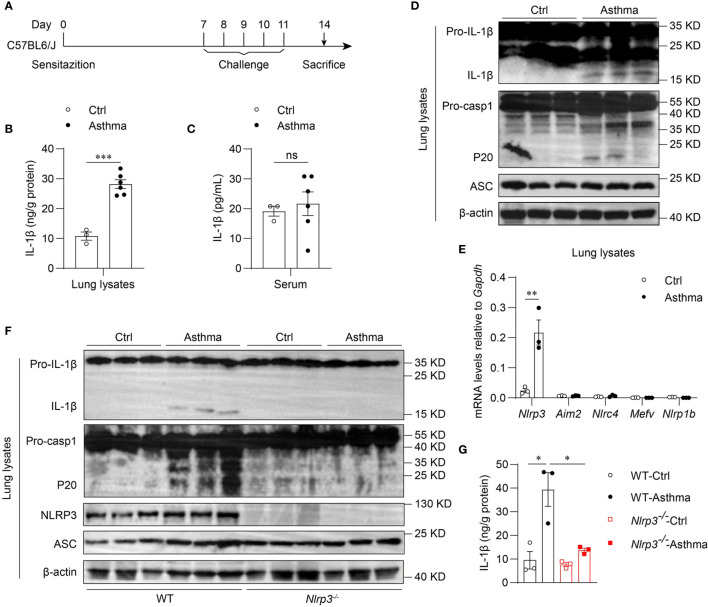
Inhaled HDM induces NLRP3 inflammasome activation in allergic asthma. **(A)** Mice were sensitized and challenged with 10μg HDM i.n. on day 0 and days 7-11. The mice in control group were sensitized and challenged by inhaling saline. On day 14, all mice were euthanized for analysis. **(B–E)** Wild type mice were stimulated with HDM or saline for 14 days. ELISA analysis of IL-1β in lung lysates **(B)** and serum **(C)**; *n* = 3-6 biologically independent mice. **(D)** Western blot analysis of IL-1β, caspase-1 and ASC in lung lysates; *n* = 2 biologically independent experiments. **(E)** *Nlrp3*, *Aim2*, *Nlrc4*, *Mefv* and *Nlrp1b* mRNA levels in the lungs were measured using qPCR; *n* = 3 biologically independent mice. **(F, G)** Wild type and *Nlrp3^-/-^* mice were sensitized and challenged with HDM. Mice were euthanized on day 14 for analysis. **(F)** Western blot analysis of IL-1β, caspase-1, ASC and NLRP3 in lung lysates; *n* = 2 biologically independent experiments. **(G)** ELISA analysis of IL-1β in WT and *Nlrp3^-/-^* mice lung lysates; *n* = 3 biologically independent mice. Statistical significances were analyzed by unpaired Student’s *t*-test: *P < 0.05, **P < 0.01, ***P < 0.001 , ns (not significant).

To determine whether the inflammasome is activated in allergic asthma, we used an HDM-induced allergic asthma model (**Figure 1A**). Bronchoalveolar lavage fluid (BALF) cells were segregated as CD45^-^ cells, eosinophils, alveolar macrophages, neutrophils, T cells, B cells and dendritic cells (**Supplementary 1A**). BALF cells from HDM-challenged mice had more eosinophils, neutrophils and lymphocytes than saline-treated control mice after 14 days of HDM treatment (**Supplementary 2A**). In contrast, alveolar macrophage counts were decreased in HDM-treated asthmatic mice (**Supplementary 2A**). After HDM stimulation, mature IL-1β in the lung lysates of asthmatic mice was significantly increased compared with that in control mice (**Figures 1B, D**). However, the IL-1β concentration in serum was not affected by HDM challenge (**Figure 1C**), indicating that HDMs induced local IL-1β secretion in the lungs rather than systemic inflammation. The expression of mature caspase-1 was also elevated in the lungs of HDM-treated mice (**Figure 1D**). Taken together, these results suggest that HDMs stimulate the lung to produce the proinflammatory cytokine IL-1β.

Five receptor proteins have been confirmed to assemble inflammasomes, including NLRP1, NLRP3, NLRC4, AIM2 and pyrin (35). We found that only NLRP3 was highly expressed in mouse lungs, and the expression of NLRP3 was significantly increased after HDM challenge (**Figures 1E, F**). Therefore, we hypothesized that the maturation of IL-1β in the lungs was dependent on the activation of the NLRP3 inflammasome in HDM-induced asthma. Next, we used HDM to induce allergic asthma in *Nlrp3^-/-^* mice and wild-type mice. The results showed that NLRP3 knockout attenuated IL-1β production in asthmatic lungs. HDM treatment induced caspase-1 cleavage and IL-1β maturation in wild-type mouse lungs, but not in *Nlrp3^-/-^* mouse lungs (**Figures 1F, G**), indicating that inflammasome activation in asthmatic lungs is dependent on NLRP3. Collectively, HDM challenge promotes the activation of the NLRP3 inflammasome in the lungs of asthmatic mice.

### HDM Challenge Induces Activation of the NLRP3 Inflammasome in Alveolar Macrophages

**Figure 2:**
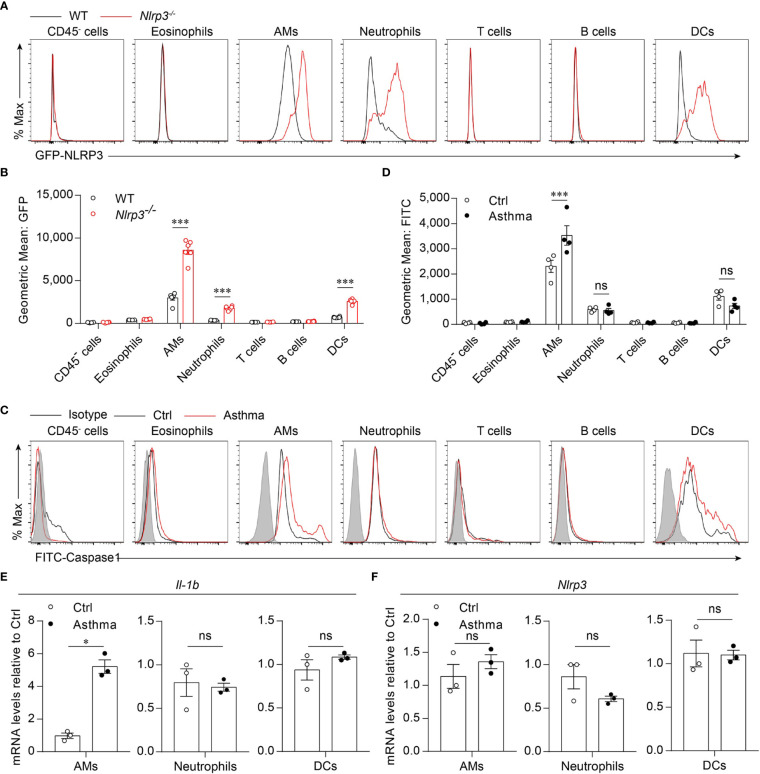
HDM induces NLRP3 inflammasome activation in alveolar macrophages. **(A, B)** Flow cytometry analysis of the GFP fluorescence intensity in the cells from the lung of WT and *Nlrp3^-/-^* mice induced allergic asthma with HDM. The histograms **(A)** and the geometric mean fluorescence intensities (Geo MFIs) **(B)** of GFP; *n* = 6 biologically independent mice. **(C, D)** Flow cytometry analysis **(C)** and quantification **(D)** of cleaved caspase-1 activation in lung cells; *n* = 4 biologically independent mice. **(E, F)** *IL-1β* **(E)** and *Nlrp3* **(F)** mRNA levels in AMs, neutrophils and DCs sorted from control and asthmatic mice lungs; *n* = 3 biologically independent mice. Statistical significances were analyzed by unpaired Student’s *t*-test: *P < 0.05, ***P < 0.001, ns (not significant).

Because we found that the activation of the NLRP3 inflammasome was induced by the HDM challenge, we next investigated which cell profile was involved in the activation of the NLRP3 inflammasome in the lungs. To detect NLRP3 expression in lung cell subsets, we used *Nlrp3^-/-^* mice in which the eGFP fluorescence intensity correlated with NLRP3 expression (33, 36). We found that in asthmatic mice, NLRP3 was expressed in the myeloid cells, including alveolar macrophages, neutrophils and dendritic cells, but not in eosinophils, T cells, B cells or CD45^-^ cells (**Figures 2A, B**). The activation of caspase-1 in lung cells was evaluated by a fluorescently labeled inhibitor of caspase (FLICA) probe, and caspase-1 activation was detected in all three cell types expressing NLRP3 during the steady state. However, in asthmatic lungs, the activation of caspase-1 was upregulated only in alveolar macrophages, while in neutrophils and dendritic cells there was no significant difference in caspase-1 activation between control and asthmatic mice (**Figures 2C, D**). We next isolated AMs, neutrophils and DCs from single-cell suspensions of healthy and asthmatic mouse lung tissue to detect the expression of IL-1β and NLRP3. Similarly, the expression of IL-1β was increased in alveolar macrophages, while it remained unchanged in neutrophils and dendritic cells after asthma was induced (**Figure 2E**). The expression of NLRP3 was not changed in alveolar macrophages, neutrophils or dendritic cells (**Figure 2F**), but was increased in asthmatic lungs because the percentage of inflammatory cells was upregulated during asthma (**Figure 1E**, **F** and **Supplementary 2A**). Treatment with HDMs did not promote caspase-1 activation or IL-1β secretion in bone marrow-derived macrophages (BMDMs) (**Supplementary 3A**). In vitro HDM stimulation did not promote inflammasome activation in isolated alveolar macrophages from wild-type mice either (**Supplementary 3B**). The reason for this might be that alveolar macrophages do not recognize HDMs directly but respond to danger signals released by damaged cells or other signals caused by HDM stimulation *in vivo*. Taken together, our results demonstrated that HDM-induced NLRP3 inflammasome activation in the lungs was dependent on alveolar macrophages.

### NLRP3 Promotes the Inflammation and the Type II Immune Response in HDM-Induced Allergic Asthma

**Figure 3:**
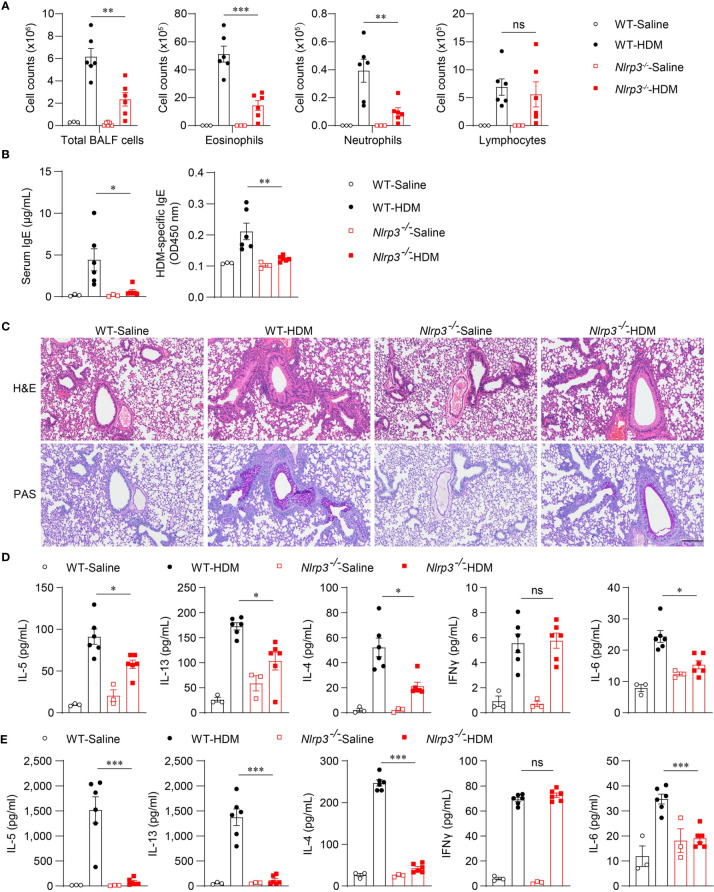
NLRP3 deficiency attenuates airway inflammation HDM-induced asthma. **(A–E)** WT and *Nlrp3^-/-^* mice were sensitized and challenged with 10μg HDM i.n. on day 0 and days 7-11. The mice in control group were sensitized and challenged by inhaling saline. On day 14, all mice were euthanized for analysis. **(A)** counts of total BALF cells, eosinophils, neutrophils and lymphocytes. **(B)** ELISA analysis of IgE and HDM-specific IgE in serum. **(C)** Histological analysis of lung sections stained with H&E and PAS (Scale bar = 200 μm); *n* = 3 biologically independent experiments. ELISA analysis of IL-4, IL-5, IL-13, IL-6 and IFNγ in BALF **(D)** and supernatants of MLN cells obtained from control or challenged mice after or without restimulation with HDM **(E)**. *n* = 3-6 biologically independent mice **(A, B, D, E)**. Statistical significances were analyzed by unpaired Student’s *t*-test: *P < 0.05, **P < 0.01, ***P < 0.001, ns (not significant).

Because the NLRP3 inflammasome was activated by HDM stimulation *in vivo*, we next investigated whether NLRP3 affected the development of allergic asthma. We used an HDM-induced asthma model in *Nlrp3^-/-^* mice and wild-type mice. Compared with wild-type mice, the counts of total BALF cells were reduced in *Nlrp3^-/-^* mice exposed to HDM (**Figure 3A**). Compared with wild-type mice, BALF from *Nlrp3^-/-^* mice contained lower counts of eosinophils and neutrophils (**Figure 3A**). Total IgE and HDM-specific IgE in the serum were also detected, revealing that *Nlrp3^-/-^* mice had decreased concentrations of serum IgE, indicating a reduced T_H_2 response and less HDM-specific B cell maturation in *Nlrp3^-/-^* mice (**Figure 3B**). Lung histology studies also showed decreased peribronchial and perivascular leukocyte infiltration in the airways of *Nlrp3^-/-^* mice after HDM challenge (**Figure 3C**). PAS staining showed that the mucus production and goblet cell hyperplasia induced by HDM exposure were diminished in *Nlrp3^-/-^* mouse lungs (**Figure 3C**). To further investigate the HDM-induced T_H_2 response, we measured cytokine secretion in BALF. BALF from HDM-treated *Nlrp3^-/-^* mice exhibited significantly less IL-4, IL-5 and IL-13 production compared with BALF from HDM-treated wild-type mice (**Figure 3D**). IL-6 production was also reduced in *Nlrp3^-/-^* BALF (**Figure 3D**). BALF from HDM-treated *Nlrp3^-/-^* mice had a comparable number of lymphocytes and IFN-γ secretion to wild-type mice, and NLRP3 impaired only the T_H_2 subset of helper T cells in HDM-induced allergic asthma. Next, we isolated cells from the mediastinal lymph nodes (MLNs) and restimulated these cells with HDMs *in vitro*. We also found reduced production of IL-4, IL-5, IL-6 and IL-13 in *Nlrp3^-/-^* MLNs, while there was no difference in the concentration of IFN-γ between HDM-treated wild-type and *Nlrp3^-/-^* mice (**Figure 3E**). Collectively, these results show that NLRP3 is required for the promotion of T_H_2 responses and the pathological damage caused by asthma.

### NLRP3 Promotes HDM-Induced Allergic Asthma in an Inflammasome-Dependent Manner

**Figure 4:**
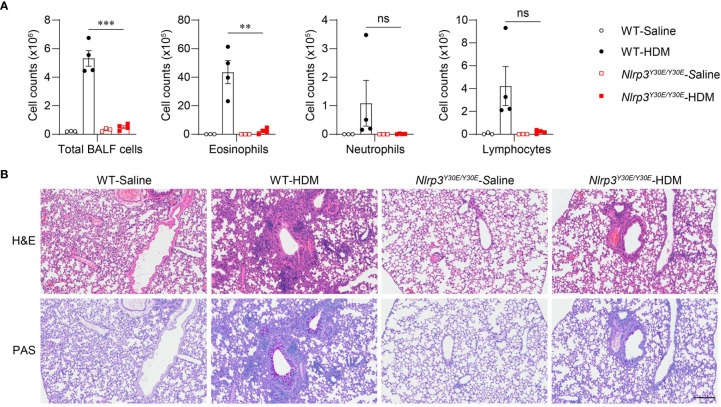
*Nlrp3^Y30E/Y30E^* mice have attenuated inflammation in HDM-induced asthma. **(A, B)** WT and *Nlrp3^Y30E/Y30E^* Mice were sensitized and challenged with 10μg HDM i.n. on day 0 and days 7-11. The mice in control group were sensitized and challenged by inhaling saline. On day 14, all mice were euthanized for analysis. **(A)** Counts of total BALF cells, eosinophils, neutrophils and lymphocytes; *n* = 4 biologically independent mice. **(B)** Histological analysis of lung sections stained with H&E and PAS (Scale bar = 200 μm); *n* = 3 biologically independent experiments. Statistical significances were analyzed by unpaired Student’s *t*-test: **P < 0.01, ***P < 0.001, ns (not significant).

To explore whether activation of the NLRP3 inflammasome is involved in the progression of asthma, we applied the HDM model in *Nlrp3^Y30E/Y30E^* mutant mice. The *Nlrp3^Y30E/Y30E^* mutation does not affect NLRP3 expression but completely inhibits the NLRP3 inflammasome activation through blockade of NLRP3 dephosphorylation at Tyr30. We found that HDM-induced eosinophil infiltration was interrupted in *Nlrp3^Y30E/Y30E^* mice, aligning with the phenotype in *Nlrp3^-/-^* mice (**Figure 4A**). *Nlrp3^Y30E/Y30E^* mice exposed to HDM showed decreased numbers of infiltrating inflammatory cells and mucus secretion compared with wild-type mice, as determined by H&E and PAS staining of airway cells (**Figure 4B**). The results demonstrated that NLRP3 promoted inflammation in asthma by forming inflammasomes. Thus, in response to HDM stimulation, NLRP3 promotes lung inflammation and pathological damage in an inflammasome-dependent manner.

### Inhibition of NLRP3 Has Therapeutic Effects on HDM-Induced Allergic Asthma

**Figure 5:**
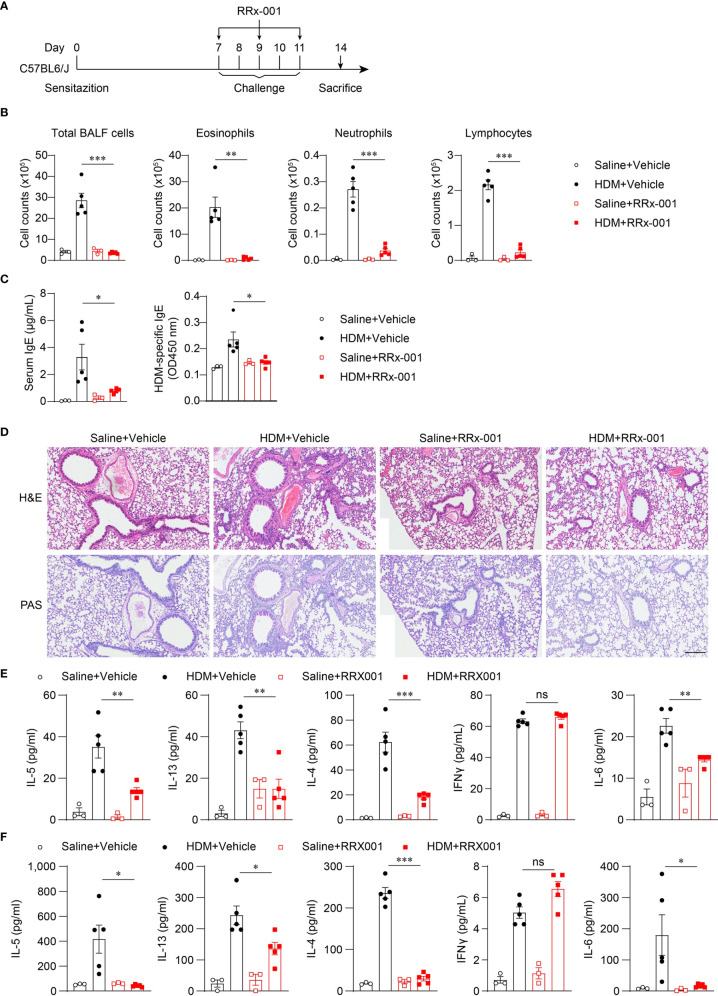
RRx-001 treatment has therapeutic effects on HDM-induced allergic asthma. **(A)** Mice were induced allergic asthma with 10μg HDM i.n. on day 0 and days 7-11, and given 10mg/kg RRx-001 or vehicle i.p. on days 7, 9, and 11. The mice in control group were challenged by inhaling saline, and given 10mg/kg RRx-001 or vehicle i.p. on days 7, 9, and 11. On day 14, all mice were euthanized for analysis. **(B–F)** WT mice were induced allergic asthma with HDM and treated with RRx-001 or vehicle. **(B)** Counts of total BALF cells, eosinophils, neutrophils and lymphocytes. **(C)** ELISA analysis of IgE and HDM-specific IgE in serum. **(D)** Histological analysis of lung sections stained with H&E and PAS (Scale bar = 200 μm); *n* = 3 biologically independent experiments. ELISA analysis of IL-4, IL-5, IL-13, IL-6 and IFNγ in BALF **(E)** and supernatants of MLN cells obtained from control or challenged mice after or without restimulation with HDM **(F)**. *n* = 3-5 biologically independent mice **(B, C, E, F)**. Statistical significances were analyzed by unpaired Student’s *t*-test: *P < 0.05, **P < 0.01, ***P < 0.001, ns (not significant).

*Nlrp3^-/-^* mice were resistant to lung inflammation in HDM-induced allergic asthma. Owing to the development of NLRP3 inflammasome inhibitors, we investigated whether the inhibition of NLRP3 *in vivo* had a therapeutic effect on asthmatic mice. RRx-001 is a highly selective and potent NLRP3 inhibitor and has beneficial effects on NLRP3-driven inflammatory diseases (37). RRx-001 (1-bromoacetyl-3,3-dinitroazetidine) is an anticancer agent currently in phase III clinical trials and is a well-tolerated agent without clinically significant toxic effects (38). Thus, we explored the effect of this safe and effective NLRP3 inhibitor on asthma. HDM-treated mice were intraperitoneally injected with RRx-001 or vehicle on days 7, 9, and 11 (**Figure 5A**), and mice were sacrificed on day 14 to detect airway inflammation. The results showed that treatment with RRx-001 (10mg/kg) significantly inhibited HDM-induced infiltration of eosinophils, neutrophils and lymphocytes in the airway and suppressed eosinophilic airway inflammation (**Figure 5B**). In contrast to vehicle-treated mice, total IgE and HDM-specific IgE in the serum were decreased in RRx-001-treated mice (**Figure 5C**). RRx-001-treated mice displayed reduced peribronchial and perivascular infiltration of inflammatory cells and lower goblet-cell hyperplasia (**Figure 5D**). The production of the T_H_2 response cytokines IL-4, IL-5 and IL-13 in the BALF and the supernatants of MLN lymphocytes restimulated with HDM *in vitro* were severely reduced in RRx-001-treated mice (**Figures 5E, F**). IL-6 production was also abrogated by RRx-001 treatment, while IFN-γ secretion was not altered (**Figures 5E, F**). These phenomena were consistent with *Nlrp3^-/-^* mice. These results suggested that the NLRP3 inhibitor RRx-001 reduced the inflammation in the lungs and had therapeutic effects on HDM-induced allergic asthma.

**Figure 6:**
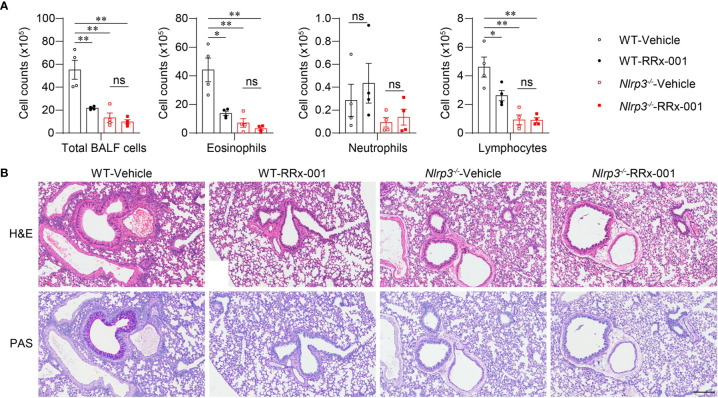
The therapeutic effects of RRx-001 on allergic asthma are dependent on NLRP3. **(A, B)** WT and *Nlrp3^-/-^* mice were induced allergic asthma with 10μg HDM i.n. on day 0 and days 7-11, and given 10mg/kg RRx-001 or vehicle i.p. on days 7, 9, and 11. On day 14, all mice were euthanized for analysis. **(A)** Counts of total BALF cells, eosinophils, neutrophils and lymphocytes; *n* = 4 biologically independent mice. **(B)** Histological analysis of lung sections stained with H&E and PAS (Scale bar = 200 μm); *n* = 3 biologically independent experiments. Statistical significances were analyzed by unpaired Student’s *t*-test: *P < 0.05, **P < 0.01, ns (not significant).

To further investigate whether the therapeutic effects of RRx-001 on asthma are dependent on NLRP3, we treated HDM-challenged *Nlrp3^-/-^* mice and wild-type mice with RRx-001 and vehicle. The experimental results revealed no significant differences in the number of eosinophils, neutrophils or lymphocytes in the BALF of *Nlrp3^-/-^* mice between the RRx-001-treated group and the vehicle-treated group (**Figure 6A**). While RRx-001 inhibited BALF cells increases in wild-type mice, it had no effect on the inflammatory cell infiltration of *Nlrp3^-/-^* mice in HDM-induced asthma. RRx-001 did not affect HDM-induced pathological lung damage or goblet cell mucus secretion in *Nlrp3^-/-^* mice (**Figure 6B**). These results demonstrated that the therapeutic effects of RRx-001 on allergic asthma were dependent on NLRP3.

## Discussion

In this study, we demonstrate that HDM challenge induced NLRP3 inflammasome activation in alveolar macrophages in the airway, which promoted lung inflammation and tissue damage in asthma. We identified the NLRP3 inflammasome as an important regulator in the development of asthma and identified an inhibitor targeting NLRP3 that is effective for treating HDM-induced allergic asthma. These findings suggest that NLRP3 in AMs is a key component in eosinophilic allergic asthma progression and indicate an effective drug targeting NLRP3 for asthma therapy.

Our results showed that NLRP3 promoted airway inflammation and tissue damage in HDM-induced eosinophilic asthma in an inflammasome-dependent manner. *Nlrp3^-/-^* mice have less eosinophil recruitment and mucus secretion in the airway. However, there are controversial results regarding the role of the NLRP3 inflammasome activity in allergic asthma. Inhaling allergens not only promotes the activation of the NLRP3 inflammasome in epithelial cells but also increases the interaction of NLRP3 and the transcriptional factor IRF4 in T_H_2 cells. By inducing an asthma model in *Nlrp3^Y30E/Y30E^*-mutant mice, which completely abrogates inflammasome activation without altering the expression of NLRP3, we demonstrated that the inflammasome activity of NLRP3 was critical for the development and progression of asthma.

It has been demonstrated that the expression of NLRP3, caspase-1 and IL-1β is enhanced in the lungs of asthma patients and allergen-induced asthmatic mice compared to healthy controls (22, 39). However, it is unclear which cell profile the NLRP3 inflammasome activates. Some research suggests that NLRP3 in bronchial epithelial cells or T_H_2 cells promotes the inflammatory response in allergic asthma (22, 29). However, NLRP3 is weakly expressed by nonmyeloid cells (33). In our study, NLRP3 expression was not detected in CD45^-^ cells, and active caspase-1 was also negative in CD45^-^ cells after HDM challenge. Instead, we found that NLRP3 was highly expressed in alveolar macrophages, neutrophils and dendritic cells in the lungs. Surprisingly, there was active caspase-1 in all cell types expressing NLRP3 during steady state, which was consistent with the IL-1β level in the lungs of healthy mice. The reason for this might be that the respiratory system is continuously in contact with external antigens, leading to constantly weak IL-1β secretion in the alveoli environment. Interestingly, during HDM-induced asthma, caspase-1 activation and IL-1β maturation were upregulated only in alveolar macrophages, not in neutrophils or DCs, indicating that AMs contributed most to HDM-induced inflammasome activation. In healthy mouse lungs the cells in the alveoli are mainly alveolar macrophages. After allergen challenge, there were more eosinophils, neutrophils and lymphocytes in the BALF, but the number of AMs was significantly decreased. The mechanism of AM reduction in asthma is unclear. Based on our results, we suspect that the pyroptosis of AMs induced by NLRP3 inflammasome activation might be the reason for AM metaplasia, but this requires further exploration.

Our results indicate that the NLRP3-specific inhibitor RRx-001 exerts beneficial effects on allergic asthma. Several NLRP3-targeted inhibitors with potential beneficial effects have been identified (40–43). Among them, RRx-001 is closer to clinical application and more effective (RRx-001 inhibited IL-1β production with an IC50 value of 116.9 nM) (37). Therefore, we selected RRx-001 to target NLRP3 in asthma, and it showed strong therapeutic effects. We surmise that RRx-001 might have a long-lasting effect because it can covalently bind to NLRP3 after administration. Subsequent research will focus on whether extended dosing intervals or low-dose inhalation administration still have therapeutic effects on asthma, which could make RRx-001 administration a more convenient medication regimen.

The pathogenesis of asthma has been difficult to elucidate, and the treatment methods have been limited. Our study demonstrates NLRP3 as an important regulator in eosinophilic allergic asthma and that RRx-001, a phase III clinical drug, can target NLRP3 to heal allergic asthma, providing new ideas for the treatment of this disease.

## Data Availability Statement

The raw data supporting the conclusions of this article will be made available by the authors, without undue reservation.

## Ethics Statement

The animal study was reviewed and approved by Animal Care Committee of University of Science and Technology of China.

## Author Contributions

MM performed most of the experiments of this work. GL and MQ performed the H&E and PAS staining experiments. WJ and RZ designed the research. MM, WJ, and RZ wrote the manuscript. WJ and RZ supervised the project. All authors contributed to the article and approved the submitted version.

## Funding

This research was supported by the National Key Research and Development Program of China (grant number 2019YFA0508503, 2020YFA0509101, 2018YFA0507403), the Strategic Priority Research Program of the Chinese Academy of Sciences (grant number XDB29030102), the National Natural Science Foundation of China (grant numbers U20A20359, 81821001, 31770991, 91742202), the Fundamental Research Funds for the Central Universities and the University Synergy Innovation Program of Anhui Province (GXXT-2019-026).

## Conflict of Interest

RZ and WJ are named as inventors on China National Intellectual Property Administration Application Serial No. 202011472140.0 related to RRx-001.

The remaining authors declare that the research was conducted in the absence of any commercial or financial relationships that could be construed as a potential conflict of interest.

## Publisher’s Note

All claims expressed in this article are solely those of the authors and do not necessarily represent those of their affiliated organizations, or those of the publisher, the editors and the reviewers. Any product that may be evaluated in this article, or claim that may be made by its manufacturer, is not guaranteed or endorsed by the publisher.
