# Supplementary material for: Inhibition of the Inflammasome Activity of NLRP3 Attenuates HDM-Induced Allergic Asthma
Source: Front Immunol. 2021 Aug 3;12:718779. doi: 10.3389/fimmu.2021.718779 (PMC8369415; doi:10.3389/fimmu.2021.718779)
Supplement: Supplementary file 1 [file Table_1.docx]

**Supplemental Information**

**Inhibition of the pro-inflammasome activity of NLRP3 attenuates HDM-induced allergic asthma**

Ming Ma, Guoyang Li, Minghui Qi, Wei Jiang, Rongbin Zhou

Correspondence to: Dr. Rongbin Zhou ([zrb1980@ustc.edu.cn](mailto:zrb1980@ustc.edu.cn)) and Dr. Wei Jiang ([ustcjw@ustc.edu.cn](mailto:ustcjw@ustc.edu.cn))

**This file include:**

Figures S1 to S3

**Fig. S1**


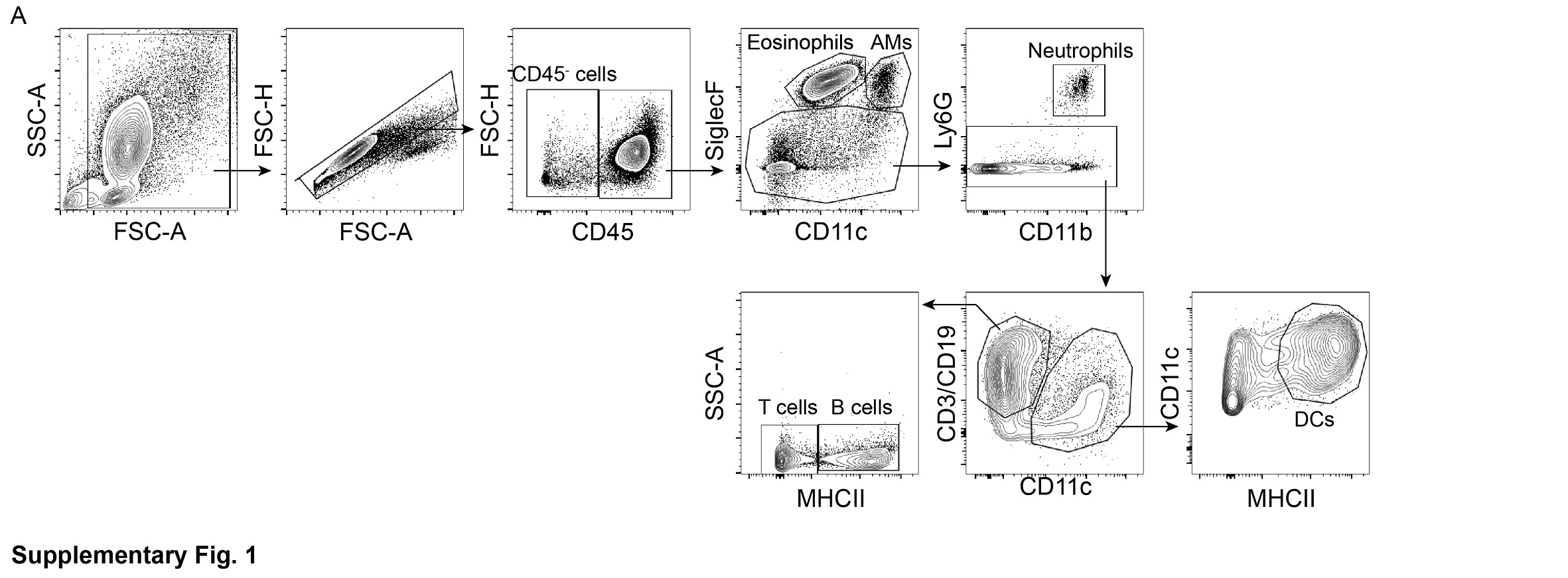


**Supplementary Figure. 1** **A**, Representative gating strategy for FACS used to quantify the ratio of CD45^-^ cells, eosinophils, alveolar macrophages, neutrophils, B cells, T cells and dendritic cells.

**Fig. S2**


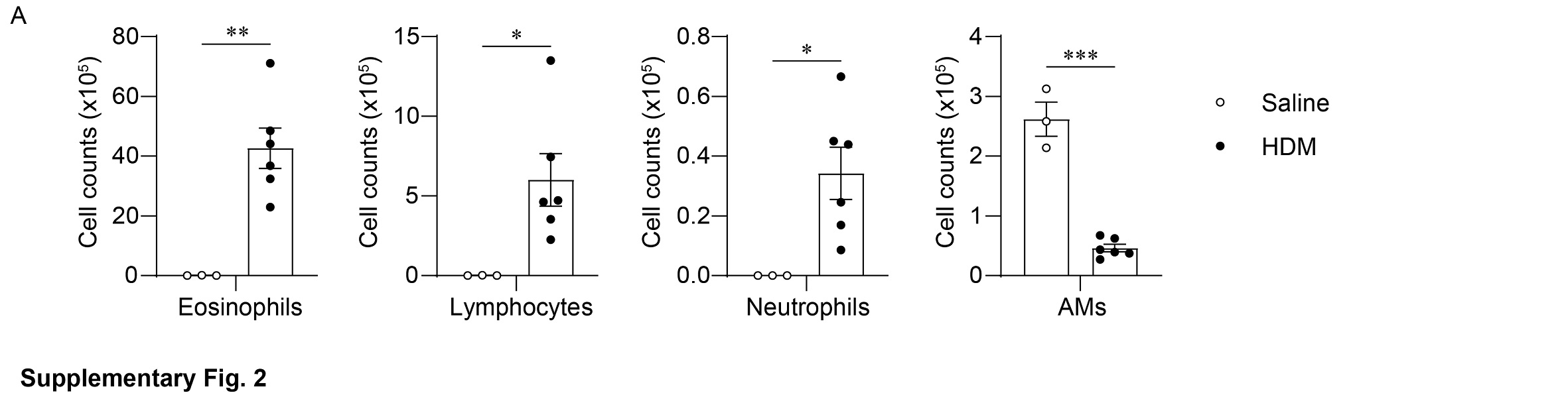


**Supplementary Figure. 2 A**, WT mice were induced allergic asthma with 10μg HDM i.n. on day 0 and days 7-11. On day 14, mice were euthanized for analysis. Counts of eosinophils, neutrophils, lymphocytes and AMs in BALF. *n* = 3-6 biologically independent mice. Statistical significances were analyzed by unpaired Student’s *t*-test: **P* < 0.05, ***P* < 0.01, ****P* < 0.001.

**Fig. S3**


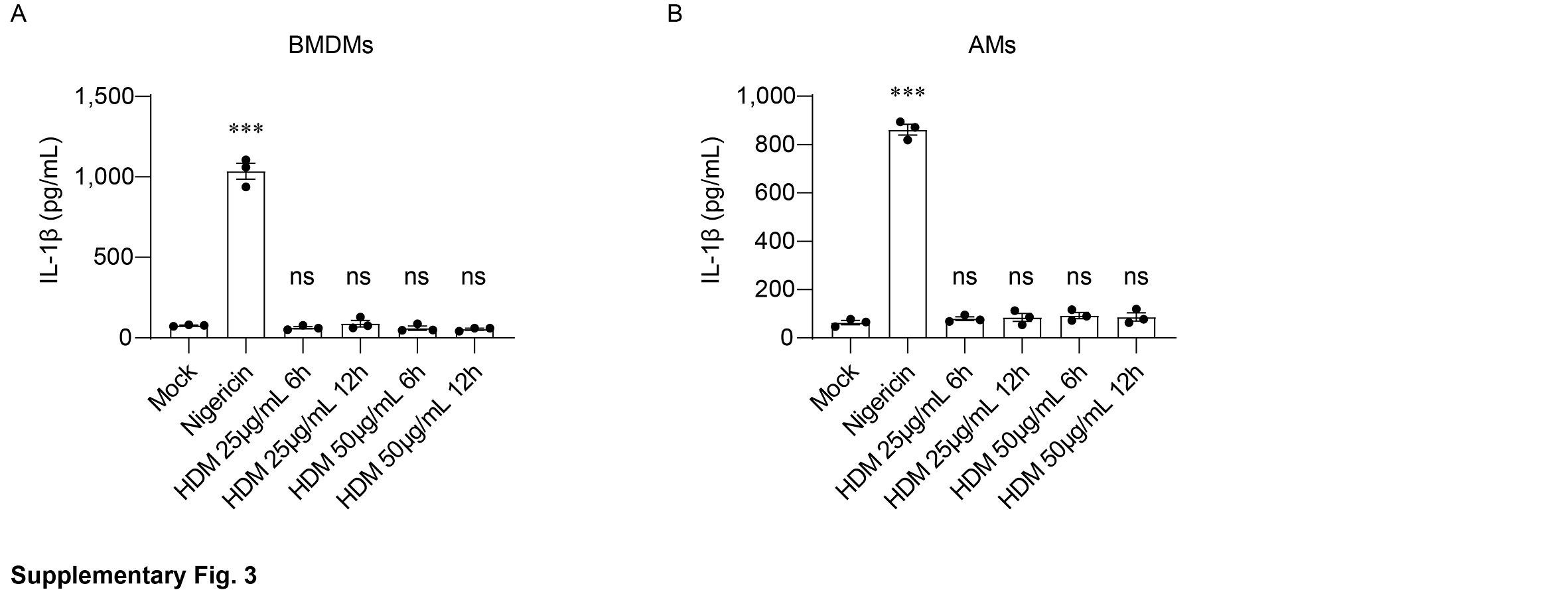


**Supplementary Figure. 3 (A,B)** ELISA analysis of IL-1β in the supernatants of BMDMs **(A)** and AMs **(B)**, primed by LPS and stimulated with Nigericin or HDM. *n* = 3 biologically independent samples. Statistical significances were analyzed by unpaired Student’s *t*-test: **P* < 0.05, ***P* < 0.01, ****P* < 0.001, ns (not significant).
